# IKKα modulates primary sclerosing cholangitis and intrahepatic cholangiocarcinoma

**DOI:** 10.1186/2051-1426-2-S3-P171

**Published:** 2014-11-06

**Authors:** Qun Jiang, Anthony J Scarzello, Zuoxiang Xiao, Timothy Back, Jami Willette-Brown, Feng Zhu, Yinling Hu, Robert H Wiltrout

**Affiliations:** 1 NCI-Frederick, MD, USA

## 

Intrahepatic cholangiocarcinoma (ICC) is the second most common primary liver cancer after hepatocellular carcinoma (HCC). The risk of ICC is higher in patients with primary sclerosing cholangitis (PSC). To date, the cellular and molecular mechanism underlying the pathological progression of PSC and ICC is poorly understood. IKKα is part of the IκB kinase (IKK) complex, which plays an important role in regulating inflammation- associated carcinogenesis through both NF-κB-dependent and independent pathways. Here, we show that IKKα mutant mice developed very serious PSC as early as four weeks of age. The ALT/AST and bilirubin levels were significantly increased in the serum of IKKα mutant mice along with lymphocytic and eosinophilic infiltration into the liver. Liver inflammation in the IKKα mutant mice, mediated by macrophages, neutrophils and CD4 T cells, was associated with the death of cholangiocytes and hepatocytes, and obstruction of intrahepatic and extrahepatic bile ducts, which impeded bile flow and ultimately led to biliary fibrosis and cirrhosis. Additionally, upon activation of NOTCH signaling in the liver via hydrodynamic shear, we observed that NOTCH-induced ICC, with the PSC, developed significantly faster in IKKα mutant mice. To identify whether intrinsic IKKα dysfunction in hepatocytes promotes the NOTCH-induced ICC in IKKα mutant mice, we generated IKKα hep KO mice, in which IKKα is conditionally knocked out in hepatocytes. No biliary disease or liver injury was observed in these mice. We then established an accelerated ICC model utilizing hydrodynamic delivery of NICD and AKT expression vectors. Unexpectedly, ICC development was remarkably slower in the IKKα hep KO mice compared to the IKKα floxed control mice, and this delayed ICC development was associated with reduced activation or levels of AKT, NOTCH, MAPK/Erk and c-myc. These data suggest that IKKα may play a protective role in PSC, while promoting ICC derived from hepatocytes. In contrast to ICC, we also established the cMET/β-catenin-induced HCC model in the IKKα hep KO mice, which did not reveal any difference in tumor development between IKKα hep KO and control mice. Taken together, our findings suggest that IKKα plays complicated and important roles in PSC and ICC pathological progression.

